# The Impact of the Moderating Effect of Psychological Health Status on Nurse Healthcare Management Information System Usage Intention

**DOI:** 10.3390/healthcare8010028

**Published:** 2020-02-02

**Authors:** Shih-Jung Hsiao, Hsiao-Ting Tseng

**Affiliations:** 1 School of Economics and Management of Guangdong Institute of Petrochemical Technology, Maoming 525000, China; benjamin@mis.ccu.edu.tw; 2 Department of Information Management, National United University, Miaoli 36003, Taiwan

**Keywords:** nurses, psychological health status, healthcare management information system, usage intention, technology acceptance model

## Abstract

Nurses play a key role in healthcare but work in a highly stressful and unfriendly environment. Therefore, many medical institutions have adopted nurse healthcare management information systems for nurses to relieve symptoms of mental stress and even improve their psychological health. The key to the success of these systems depends on how nurses intend to use them. In this study, the moderating effect of nurses’ psychological health status on their usage of these systems are discussed. This study used a mail survey method for nurses to obtain 1565 valid samples. The results show that perceived usefulness is insignificant toward the usage intention of nurses with a positive psychological health status, which indicates that this system does not meet the needs of these healthy nurses. Furthermore, perceived ease of use is insignificant toward the usage intention of nurses with a negative psychological health status, which indicates that a negative psychological health status may affect one’s behavior due to impatience. This study raises the serious issue that nurses should maintain their psychological health in order to ensure the quality of care for patients. People in various fields are expected to pay attention to the psychological health status of nurses and create a win–win situation for both patients and nurses.

## 1. Introduction

Nurses play the most important key role in healthcare. Their role is to actively avoid mistakes; for example, mistakes must not be made regarding drugs and injections, as any mistakes in this area will cause patient dissatisfaction and even affect the patient’s life. Thus, most nurses’ psychology is in poor condition. Nurses’ work is carried out continuously and in a highly stressful working environment, which negatively impacts their psychological health status (excluding incompetent nurses with psychological illnesses). Although the poor status of nurses’ mental health does not affect their daily life and work, it is an invisible occupational hazard accumulated over a long period of time, and the impact on one’s overall health is long-term [1,2]. The World Health Organization (WHO) has set World Mental Health Day to call on the public to pay attention to the comprehensiveness of physical, mental, and social life in order to achieve complete health. In order to achieve comprehensive health and to improve the current lack of psychological healthcare for nurses, many medical institutions have adopted healthcare management information systems (HMIS) for nurses to relieve the symptoms of deteriorating psychological health and to even improve mental health. 

HMIS is one of several health information technologies (HITs). It is the application of information processing, involving both computer hardware and software that deals with the storage, retrieval, sharing, and use of healthcare information, data, and knowledge for communication and decision making [3]. It can promote the exchange of health information between patients and healthcare service providers through the Internet. HMIS can encourage patients to share their health information with their healthcare providers by increasing the mutual cooperation in healthcare, as well as eliminating medical errors, improving healthcare efficiency, and reducing costs; furthermore, it can improve the quality of healthcare [4,5]. HMIS can be regarded as a service interface through the application of smart phones to provide the public with a combination of professional medical sorting, personalized self-management, grading tracking, complex e-health promotion, and disease management services. Furthermore, because of the work required, HMIS is often accompanied by pressure from environmentalist groups. Although HMIS has a wide range of potential positive benefits and assists individuals with self-health management, many studies and related reports have found that many people never actually use HMIS. Although its benefits have a keen interest, even in the United States, which includes a health management system, there are still not many users. The concept of HMIS was introduced in 1978, and until now, research has been rare, and the factors affecting the willingness to use HMIS have not been fully established [6]. Compared to other information systems, it is almost completely dependent on the user’s own wishes [6]. For the system to be truly effective and successful, it requires people’s willingness to use it. Therefore, the key to the success and effectiveness of nurse healthcare management information systems (NHMIS) depends on the nurse’s willingness to use them.

Many previous studies employed the technology acceptance model (TAM) to discuss users’ usage intentions. Davis [7] revised the structure and causality of theory of reasoned action (TRA), and developed TAM to explain and predict user acceptance of information technology, as well as to explain personal acceptance of and behaviors surrounding information technology through user perception, attitude, and intention with external variables [8]. Because the theory and measurements are not determined, only the “attitude” dimensions are maintained in TAM. It employs “usage intention (UI)” to affect the real behavior of “use”, and added “perceived ease of use (PEOU)” and “perceived usefulness (PU)”to realize users’ adoption of information systems. In 2000, Venkatesh and Davis [9] proposed the technology acceptance model 2 (TAM2) based on four long-term empirical studies. TAM 2 is based on TAM, adding “subjective norm (SN)”, which is discarded in the original model. In TAM 2, SN is considered to directly affect users’ intention of using information systems, and is related to social influence, job relevance, output quality, and result demonstrability in the cognitive instrument process. Furthermore, these variables indirectly affect the user’s usage intention through PU. In addition, “experience” is added in TAM 2, which moderates the relationship between SN, PU, and UI. “Voluntariness” is also added to moderate the relationship between SN and UI. The purpose of TAM2 is to extend PEOU and PU in TAM to explore extra determinants, and to understand how these determinants affect the user’s experience. Venkatesh and Bala [10] integrated six external variables proposed by TAM2 and Venkatesh [11] to strengthen the PEOU, such as computer self-efficacy, perceptions of external control, computer anxiety, computer playfulness, perceived enjoyment, and objective usability, and proposed the technology acceptance model 3 (TAM3). Technology acceptance model 3 (TAM3) focuses on individual IT use and adoption prediction. The development processes of TAM, TAM 2, and TAM 3 are not established according to the medical environment and its characteristics. If adopted for healthcare, the selected variables should be adjusted [12] in order to increase TAM’s appropriateness [13]. 

In addition, research also indicates that nurses’ different psychological health statuses may influence their intention and perception to NHMIS usage [14]. Thus, in this study, the moderating effect of nurses’ different psychological health statuses toward their usage intention of nurse healthcare management information systems is discussed. In conclusion, the research has three core purposes: (1) To understand NHMIS usage intention of nurses and its determinants. (2) To explore nurses’ different psychological health statuses on NHMIS usage intention impact. (3) Compared with the public, nurses have more medical knowledge. Because of this, nurses often think that they also know their own health status. However, due to the nature of nurses’ work, negative psychological health statuses are prevalent, which not only affects their own health, but also reduces the quality of nursing care. Therefore, the focus of this study is whether the psychological health status of nurses will affect their intention to use NHMIS, so as to understand the influencing factors of NHMIS usage intention in different psychological health statuses, and then improve nurses’ willingness to use NHMIS for their health self-management assistance.

## 2. Theoretical Framework, Hypotheses, and Rationale 

Among the theories of “intention to use” in the traditional information management field, TAM or its modified model (e.g., TAM2, TAM3) are stronger than other models. The advantages of TAM include its simplicity and that it is easy to understand, with information technology features, a strong theoretical foundation, and sufficient empirical support [15,16,17,18,19]. The above TAM 2 is included in the cognitive aspects of behavior and mental factors (e.g., subjective norm). It can construct a more complete model of technology acceptance when exploring the personal technology acceptance model.

Furthermore, the development process of TAM is not established according to the medical environment and its characteristics; if it is to relate to healthcare and a mobile phone setting, then the selected variables should be adjusted [12]. The scope of TAM puts more emphasis on the use of medical information systems in hospital clinical staff for HIT discussing [13]. This study also employed nurses’ psychology health status as a moderator to explore nurses’ HMIS usage intention.

**Figure 1:**
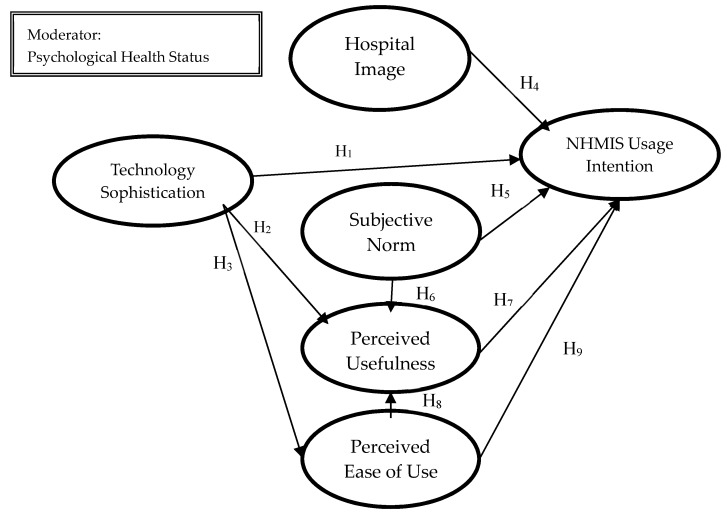
Research Framework. NHMIS, nurse healthcare management information system.

The research framework consists of the following constructs: (1) technology sophistication, (2) hospital image, (3) subjective norm, (4) perceived usefulness, (5) perceived ease of use, and (6) HMIS usage intention. According to previous research, technology sophistication affects people’s usage willingness toward electronic personal health records (ePHRs) in America [19]. Hospital image is added in this study due to findings from previous research in corporate image. Subjective norm, perceived usefulness, perceived ease of use, and HMIS usage intention are mainly derived from the extensional TAM. All of the constructs are hypothesized to affect the intention to use health management information systems. In the past, the relative evidence for the research on TAM shows that the significance among constructs relationships is inconsistent and varies between research environments. Therefore, it is considered that there are still moderators that previous research has not yet found. In the healthcare environment, nurses receive a lot of pressure from their routing operation and it may affect their job and life performance, learning quality, and even endanger their care patients [1,2]. Due to above reasons, in this study, we use psychological health status as a moderator to indicate that different psychological health statuses may affect usage intention. The research framework is shown in Figure 1.

The hypotheses in this study were obtained in line with the results of previous publications significantly addressing the concerns related to the subject of this study. Most of them are highly related to human perceptions, which are usually dependent on personality and subjectivity.

### 2.1. Technology Sophistication

The studies related to technological acceptance and innovation always emphasize the people who are equipped with higher technological knowledge and tend to have more positive attitudes toward new technology [20,21]. According to social cognitive theory [22], its core concept is self-efficacy, an individual’s perceived ability to carry out a certain action [22]. Presumably, the higher the self-efficacy, the more likely an individual is to perceive a task as effortless. Previous studies have found Internet self-efficacy to be a significant predictor of PEOU [23,24,25]. Correspondingly, Internet self-efficacy has also been found to have a significant influence on confirmation and perceived usefulness in the Internet buying context [23,26,27]. In this study, the definitions of the constructs of technological sophistication and internet self-efficacy are similar. In light of the aforesaid findings, the following hypotheses are proposed:

**Hypothesis** **1 (H1).** *Nurses’ technological sophistication positively affects their HMIS usage intention.*

**Hypothesis** **2 (H2).** *The higher the technological sophistication, the more the perceived usefulness toward HMIS.*

**Hypothesis** **3 (H3).** *The higher the technological sophistication, the more the perceived ease of use toward HMIS.*

### 2.2. Hospital Image

Hospital image is very important in accessibility of hospital services to consumers in hospital choices, as well as the role of the hospital’s image, its physical appearance, and technological capabilities in informing such choices. It is thus not created by a hospital itself, but rather by the patients and/or the public, representing their overall impressions and perceptions of the services, reputation, and characteristics of the institution [28]. 

When nurses have medical needs, they can be regarded as customers, or even more as professional customers, so when they choose whether to use HMIS, they pay more attention to Hospital image than the general public. In light of the aforesaid findings, the following hypothesis is proposed:

**Hypothesis** **4 (H4).** *Hospital Image positively affects nurses’ HMIS usage intention.*

### 2.3. Subjective Norm

Subjective norm is defined as the degree to which an individual believes that people who are important to her/him thinks he/she should perform the behavior in question [29]. In the technology domain, both peer and superior influences have been shown to be strong determinants of subjective norm [21].

Venkatesh and Davis proposed an extended model of TAM, TAM2, which includes “subjective norm”. Subjective norm has been examined in a number of studies focusing on the acceptance of technology [3,9,20,21,30]. However, TAM2 examined their effects only on “perceived usefulness”, instead of incorporating them into the nomological network of TAM. Previous studies have found that the “subjective norm” perceived by a physician will significantly influence his/her “behavioral intention”. Therefore, subjective norm can construct a more complete model of technology acceptance when exploring the personal technology acceptance model. In light of the aforesaid findings, the following hypotheses are proposed:

**Hypothesis** **5 (H5).** *The higher the nurses’ subjective norm, the more HMIS usage intention.*

**Hypothesis** **6 (H6).** *The higher the nurses’ subjective norm, the more perceived usefulness toward HMIS.*

### 2.4. The Original Technology Acceptance Model

TAM was developed by Davis in 1986. Davis developed TAM by building upon an earlier theory [31], the Theory of Reasoned Action (TRA) by Fishbein and Ajzen [29]. The purpose of TAM is “to provide an explanation of the determinants of computer acceptance that is general, capable of explaining user behavior across a broad range of end-user computing technologies and user populations, while at the same time being both parsimonious and theoretically justified” [31]. TAM has been tested widely with different samples in different situations and has proven to be a valid and reliable model in explaining acceptance of new technology [21,32,33,34].

The most significant belief of the most common version of TAM considers that determining one’s attitude toward using system, use intention, and actual use situation [31], TAM supposes that the personal acceptance of the information system is affected by two variables, including perceived usefulness (PU) and perceived ease of use (PEOU). PU is defined as thinking that the use of an information system can increase his or her working performance in subjective ways, and PEOU relates to a certain system being easy to use [31], and Attitude Toward Using (ATT) refers to a person’s positive or negative feelings of the performance of target behaviors.

TAM has 30%–40% of the explanatory power in the intention to use [21,29,32], and the PU and PEOU scales still remain strong psychological measurement characteristics [35]. Some scholars use meta-analysis to analyze TAM and confirm that PU is obviously the dominant variable of the whole model [5,15,36,37], and there are high correlations between perceived usefulness and behavior intention to use or attitude [32,38,39,40].

Davis et al. [41] found in their study that to take the personal PU and PEOU of the system as the independent variables and UI as the dependent variable to conduct a regression analysis, and the regression coefficients of the abovementioned two independent variables have no significant change as the variable of use attitude is added. Hence, the use attitude cannot completely affect the personal beliefs of the system (PU and PEOU) and the relationship between personal beliefs and usage intention (INT). Therefore, in order for the model to more simply explain INT, the final conceptualized model of TAM does not contain use attitude. Considering the aforesaid findings, the following hypotheses are proposed:

**Hypothesis** **7 (H7).** *The more the perceived usefulness for nurses, the higher their usage intention.*

**Hypothesis** **8 (H8).** *The more the perceived ease of use for nurses, the higher perceived usefulness.*

**Hypothesis** **9 (H9).** *The more the perceived ease of use for users, the higher their usage intention.*

### 2.5. Psychological Health Status

The Chinese Health Questionnaire (CHQ-12) was developed from the General Health Questionnaire (GHQ) and it has good internal consistency [42], and has been widely applied in Taiwan and China [43,44]. GHQ was started by Goldberg [45] in the 1970s and was used to assist family physicians to detect general patients without mental disorders as a screening tool of self-management, so it was also widely used by other scholars in hospitals and communities [46,47,48]. The majority of researchers take the assessment of Chong and Wilkinson [49] as the principle, that is to say, classifying the respondents whose scores >4 as the people who have poorer health statuses [50,51]. Therefore, this study regards the respondents who score >4 as the people of Psychological Health statuses.

Furthermore, the people who are more involved in self-health management should have higher evaluations on the self-health management information platform (such as e-PHR). Markle Foundation, in their survey, found that chronic patients, heavy healthcare users, and people who care for elderly patients are more interested in the personal health records (PHR) system, and chronic patients and severely ill patients have higher evaluations on a lot of descriptions of the PHR system. Furthermore, based on the description of behavioral intention in the Health Belief Model (HBM), there is a cascade of effects, starting from health status (age, disease, etc.) to perceived threat, perceived usefulness, attitude, and, finally, behavioral intention [52]. Agarwal and Angst also found in their study that chronic patients consider that the PHR system is helpful for healthcare providers, and is able to maintain closer relationships and communications [53]. Therefore, in this study, variable nurses’ psychological health status is used as a moderator in the full environment.

**Hypothesis** **10 (H10).** *Nurses’ usage intention and perception toward NHMIS varies by their psychological health status.*

## 3. Methods

### 3.1. Design and Participants

This study used systematic sampling. Empirical data for assessing the hypotheses of this study were collected by survey method. This method has been employed frequently in the social sciences field [54]. Respondents were nurses who volunteered and were providing services in medical institutions. Those who left or were involved in the future were not included in the study, and samples were collected anonymously. Nurse samples were collected from two regions (Northeast of China and Taiwan) with different cultural backgrounds [55] but frequent interactions because of similar geographic locations [56] to minimize sample bias. The medical policy in China is roughly imitated by Taiwan [57]. However, due to the differences in political beliefs between the two regions, a comparison of the development of cross-cultural studies has proven invaluable in shedding light on managerial differences in such diverse areas such as values, attitudes, and decision making. National character can be viewed as a broader explanatory level or element in the psychological makeup of decision-makers, which is reflected in consumer decision-making. Therefore, this study compared nurses of these two regions to realize their opinion toward NHMIS.

In this study, questionnaires were collected by mail survey. Data from nurse samples were collected in 2013 with research instruments based on the research framework. The minimum sample amounted to the number of questionnaires. There were 38 questions in this study, thus, it was encouraged to have at least 380 samples for further data analysis. The working style of nurses and their attitude toward questionnaire responses mentioned in previous studies were considered. This study sent more samples in the hope that the number of respondents would be greater than 380. Finally, a total of 2000 questionnaires were sent, and 1612 were collected, totaling a response rate of 80.6%. Excluding invalid samples such as incomplete answers and incorrect answers, finally, 1563 valid samples (78.15%) were collected.

In the process of sending research questionnaires, to eliminate any concerns, the cover letter clearly explained the aim and methods, and outlined the authors’ intention to publish the research as an academic journal article. In addition, this study was also sent to the Institutional Review Board for ethical approval to ensure full protection for the research subjects.

### 3.2. Questionnaire Design, Expert Panel, and Pilot Test 

The instrument design procedure was as follows: (1) A draft of the questionnaire in English was designed by referring to the measurements of related studies and then translated into Chinese. (2) In order to increase content and validity of the questionnaire, the translation accuracy was then refined and verified. Five experts who had practical and academic experience with computer systems in healthcare created an expert panel to make ensure quality. (3) To measure the constructs of the proposed framework, operational definitions and references of the questionnaire are listed in Table 1. The selected items in the instrument for each construct were mainly adapted from previous studies to ensure content validity. The items used were thus regarded to be the most appropriate because they were validated in the context of the Internet. After carefully developing the questionnaire for gathering data for the constructs investigated, the questionnaire used in this study included two parts. The first part aimed to investigate the constructs affecting intention to use HMIS. Items were adapted from the existing literature for measuring six constructs: technology sophistication, hospital image, subjective norm, perceived usefulness, perceived ease of use, and HMIS usage intention. The second part was designed to collect demographic data regarding respondents. (4) In the pilot test stage, the study invited 30 nurses to conduct a pilot test to ensure the clarity and objectivity of the questionnaire content in this study. 

### 3.3. Measures

In addition to the construct of personal characteristics, a Likert Scale was used in this study, as it is the most commonly used measure in scale design, with 5-point and 7-point Likert scales generally enjoying the largest popularities. However, Berdie [62] addressed this questionnaire design and defended the 5-point Likert scale for the following three reasons. First, in most cases, a 5-point Likert Scale is the most reliable measuring method. Once the questions are over five, it is hard for people to distinguish the right point. Secondly, the use of a 3-point Likert Scale depresses people’s strongest and mildest opinion, while a 5-point Likert Scale can express them ideally. Thirdly, a 7-point Likert Scale causes confusion for those people with poor distinguishing ability. Hence, this study adopted a 5-point Likert Scale, with the responses rated as follows: 1 as strongly disagree, 2 as disagree, 3 as somewhat agree, 4 as agree, and 5 as strongly agree.

### 3.4. Data Analysis Tools 

The study used Statistical SPSS 22.0 (SPSS Inc., Chicago, IL, USA) to conduct statistical analyses, including demographic data, descriptive results, reliability, and correlation. Moderating effect analyses were conducted by SPSS AMOS 21.0. Structural Equation Modeling (SEM) was employed to test the research hypotheses. For this study, the use of SEM provided a holistic view of how each variable directly and indirectly affects individuals’ behavioral intentions.

## 4. Results

### 4.1. Descriptive Results

The total sample size was 1565, including 141 (9%) samples collected from Taiwan and 1422 (91%) samples collected from the Northeast of China. Among them, 19 were male (1.2%) and 1546 were female (98.8%). The average age in Taiwan was 37.15 years and the average age in the Northeast of China was 30.10 years. Regarding education background, 16 nurses graduated from high school (1.1%), 663 from college (42.4%), and 856 from university (54.6%), and 30 nurses were classified as graduates (1.9%). Of the total sample size, 864 (55.2%) nurses were married and 701 were single (44.8%). When talking about their psychological health status, 1133 nurses (73.0%) reported a positive psychological health status and 432 nurses (27.0%) reported a negative psychological health status. Furthermore, we also validated Chinese and Taiwanese nurses and found that they had no significant difference.

Table 2 shows that nurses’ intention and perception have significant differences by their psychological health status; nurses with a negative psychological health status have higher degrees of PU, PEOU, SN, HI, TS, UI and PHS than nurses with a positive psychological health status. Additionally, Nurses’ HI and TS have significant differences in their marriage status.

### 4.2. Reliability Test Results

In terms of reliability analysis, the Cronbach’s Alpha Coefficient of each variable in the scale designed in this study was above 0.7, which shows that this research scale has a high degree of credibility, as shown in Table 3.

### 4.3. Test of the Hypothesized Model

**Figure 2:**
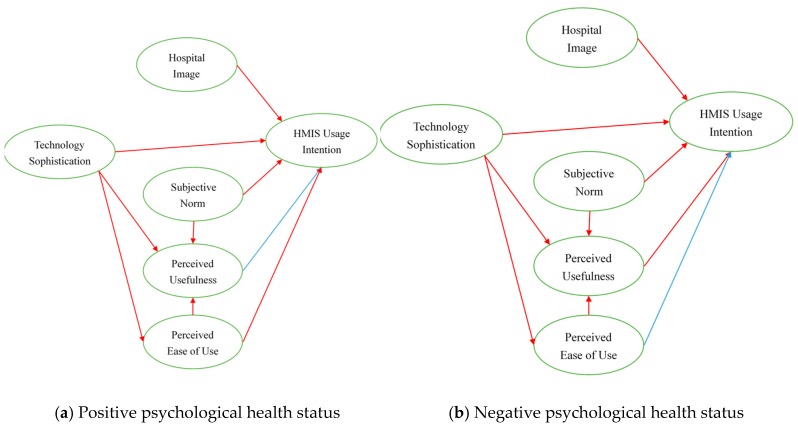
Hypotheses test results.

After the hypotheses tests, it was found that both paths were significant in the two kinds of psychological health status; therefore, Hypotheses 1, 2, 3, 4, 5, 6, and 8 are supported by data. It is worth noting that in Hypothesis 7, data only supported the hypothesis in bad psychological health status. Additionally, in Hypothesis 9, data only supported the hypothesis in good psychological health status. This shows that in Hypotheses 7 and 9, nurses’ psychological health statuses play a moderating rule in the two relationships, as shown in Table 4 and Figure 2.

Positive psychological health status:

## 5. Discussion 

In this study, the final overall explanatory power (R^2^): For nurses with a good psychological health status, the R^2^ is 0.58, and for nurses with a bad psychological health status, the R^2^ is 0.52. They both have high explanatory power. Kim and Park investigated extended TAM in the HIT arena, and found it valid to describe health consumers’ behavioral intention [63]. The study categorized the concepts in the extended TAM into three domains: health zone, information zone, and technology zone [63], which are almost the same as in this study. Differences only exist regarding the selected variables, but the discussion of these three domains will be able to achieve the research purposes of this study.

In previous research, health status may affect PU [52,64]. In addition to the health belief model (HBM) being a cognitive theory, it is also a very important value-expectancy theory. The concepts of value and expectation were reformulated in the context of health-related behavior, and the interpretations were as follows: (1) the desire to avoid illness or to get well and (2) the individual’s estimate of personal susceptibility, severity of an illness, the likelihood of being able to reduce that threat through personal action, and the belief that a specific health action available to a person would prevent illness [65].

This research adopted CHQ-12 to measure nurses’ psychological health status. However, based on research findings, the perceived usefulness of HMIS for nurses with bad psychological health status influences their HMIS usage intention. However, this relationship does not exist in nurses with good psychological health status; otherwise, perceived ease of use in nurses with a good psychological health status influences their HMIS usage intention. As per Hypothesis 7, this relationship does not exist in nurses with bad psychological health status.

In the information zone, the factors have a similar cascade effect to that in the health zone. Perceived usefulness is significantly sensitive to subjective norms. According to the theory of planned behavior (TPB), behavioral intention is a direct determinant of behaviors, arguing that subjective norm and perceived behavioral control of the action are the most powerful predicting factors for behavioral intention [29]. Previous studies have found that the subjective norm perceived by a person significantly influences his/her behavioral intention. This research supposes that subjective norms (e.g., TPB) in HIT researches have significant influence on PU and UI. In fact, previous researches also prove this result [63,66,67]. Therefore, this study also assumed that subjective norms affect PU and INT.

The results show that most of results in this study are in line with previous research hypotheses, but slightly different due to two kinds of psychological health status. Nurses with good psychological health status can clearly and subjectively distinguish a system’s effectiveness and efficiency in their operation assistance. So, for those with good psychological health status, if they consider the system useful to them, they are willing to use it in their operation process. Conversely, if nurses have bad psychological health status, they suffer mental illness which makes it difficult to clearly and subjectively realize a system’s effectiveness and efficiency, and might result in a lack of interest to adopt new technology in their routine operations, because, for them, it is difficult to become accustomed to and learn a new technology; therefore, they would not use it due to its perceived usefulness. 

Furthermore, nurses with bad psychological health status suffer mental illness which makes it difficult to clearly and subjectively realize a system’s effectiveness and efficiency. In this moment, if a user-friendly system assists them in conquering these troubles to make their routine job more effectiveness and efficiency, then they are willing to use it in their routine operations. Conversely, nurses with good psychological health status can easily to do their job perfectly; therefore, they are less interested in becoming accustomed to and learning a new system in their routine operations. For them, to become accustomed to and to learn a new system brings work burdens. In their view, they think they can do the job perfectly without the assistance of HMIS. 

In addition, this study also employed hospital image as an information zone of one variable. The concept of hospital image comes from corporate image. In research practice, corporate image has long been considered a critical factor that differentiates the products/services of one corporation from those of others, and thus it can significantly influence customers’ corporate image intentions [60]. In the healthcare field, hospital image is defined as the sum of the beliefs, ideas, and impressions of patients and/or the general public with regard to a hospital, which are developed based on their past experiences with the hospital [60,68]. Hospital image is also multidimensional; it is associated with various features from their own medical examination and treatment experiences [69] such as equipment and facilities, employee attitudes and behavior, and communication styles, and is not absolute but relative to that of competing hospitals [68]. 

The last domain is the technology zone, which has factors with the following characteristics. The technology zone in this study consisted of two parts, one was developed from the Original TAM, including the two variables of PEOU and PU, and the other was the study of the intention to use ePHR developed by Angst and Agarwal [6], including the three variables of technology sophistication, Interest in Reviewing Health Data, and Conveniences, which can jointly affect the intention to use HMIS.

## 6. Conclusions

In contrast to nurses with bad psychological health status, those with positive psychological health status perceived that usefulness is insignificant toward nurses’ usage intention, which may indicate that this system does not meet the needs of nurses with positive psychological health status. Because they do not show signs of sub-health, they do not need this system to assist them. Compared to other nurses, perceived ease of use of this system is insignificant in the negative psychological health status of these nurses toward their usage intention. 

This indicates that one of the characteristics of people with negative psychological health status is that they are more likely to be impatient, and so psychological health status is very important for a person’s life and health, especially affecting one’s explicit behavior. Especially for those nurses who care for patients in the clinical frontline, their psychological health status is a serious matter. 

Therefore, this study raises the following serious issue, that nurses should maintain their psychological health to ensure quality of care for patients. The importance of this issue has never been mentioned, and it can be said to be the focus of less attention. Therefore, this is a contribution of this research. It is hoped that people in various fields pay attention to the psychological health status of nurses and create a win–win situation for patients and nurses.

## Figures and Tables

**Table 1:** Research Operational Definition.

Construct	Operational Definition	# of Items	Researches
Psychological health status	Employed the Chinese Health Questionnaire (CHQ-12) to measure nurses’ psychological health status	12	[58,59]
Technology sophistication	Having an active email account, the number of online activity, and online count (e.g., shopped or purchased something on an online auction, ordered medications, or managed prescriptions) were included to assess ability to use information	6	[6]
Hospital image	The sum of the beliefs, ideas, and impressions of patients and/or the general public with regard to a hospital, which were developed based on their past experience with the hospital	4	[60]
Perceived usefulness	The degree to which a nurse believes that the use of HMIS would enhance his or her health	5	[31,41]
Perceived ease of use	The degree to which a nurse believes that the use of HMIS would be free of effort	4	[31,41]
Subjective norm	The degree to which a nurse believes that people who are important to her/him thinks he/she should perform the behavior	4	[20,61]
HMIS usage intention	Nurses’ intention to use NHMIS	3	[21]

**Table 2:** Demographic data.

Demographic	Category	Taiwan (*n* = 142)	Northeast of China (*n* = 1422)	F-Value						
				PU	PEOU	SN	HI	TS	UI	PHS
Gender	male	2	17	0.196	0.839	0.465	0.315	1.191	0.749	1.193
	female	140	1406							
Education background	high school	1	15	0.648	1.583	0.787	2.298	1.471	1.212.	2.403
	college	45	618							
	university	74	782							
	graduate	22	8							
Marriage status	married	88	776	2.190	0.588	2.100	2.431 *	2.089 *	1.755	0.010
	single	54	647							
Psychological health status	negative	47	385	3.908 * n > *p*	2.222 * n > *p*	3.605 * n > *p*	3.463 * n > *p*	2.668 * n > *p*	4.088 * n > *p*	2.980 * n > *p*
	positive	95	1038							

* Represents *p*-value of hypotheses <0.05. PU, perceived usefulness; PEOU, perceived ease of use; SN, subjective norm; HI, hospital image; TS, technology sophistication; UI, usage intention; PHS, psychological health status.

**Table 3:** Research Operational Definition.

Variables	Cronbach’s Alpha
Perceived Ease of Use	0.917
Perceived Usefulness	0.923
Subjective Norm	0.912
Hospital Image	0.920
Technology Sophistication	0.921
NHMIS Usage Intention	0.936
Psychological health status	0.771

**Table 4:** Hypotheses tests.

Hypotheses	Full Sample (*n* = 1563)	Positive Psychological Health Status (*n* = 1146)	Negative Psychological Health Status (*n* = 417)
H1	0.28 *	0.27 *	0.29 *
H2	0.15 *	0.11 *	0.24 *
H3	0.60 *	0.61 *	0.54 *
H4	0.49 *	0.50 *	0.44 *
H5	0.37 *	0.39 *	0.35 *
H6	0.62 *	0.68 *	0.56 *
H7	0.10 *	0.03	0.18 *
H8	0.30 *	0.44 *	0.30 *
H9	0.07 *	0.16 *	−0.08
R^2^	0.56	0.58	0.52

* Represents *p*-value of hypotheses < 0.05.
